# Structural Basis of the Interaction of a *Trypanosoma cruzi* Surface Molecule Implicated in Oral Infection with Host Cells and Gastric Mucin

**DOI:** 10.1371/journal.pone.0042153

**Published:** 2012-07-31

**Authors:** Cristian Cortez, Nobuko Yoshida, Diana Bahia, Tiago J.P. Sobreira

**Affiliations:** 1 Departamento de Microbiologia, Imunologia e Parasitologia, Escola Paulista de Medicina, Universidade Federal de São Paulo, São Paulo, São Paulo, Brasil; 2 Laboratório Nacional de Biociências, Centro Nacional de Pesquisa em Energia e Materiais, Campinas, Brasil; Centre National de la Recherche Scientifique, France

## Abstract

Host cell invasion and dissemination within the host are hallmarks of virulence for many pathogenic microorganisms. As concerns *Trypanosoma cruzi*, which causes Chagas disease, the insect vector-derived metacyclic trypomastigotes (MT) initiate infection by invading host cells, and later blood trypomastigotes disseminate to diverse organs and tissues. Studies with MT generated in vitro and tissue culture-derived trypomastigotes (TCT), as counterparts of insect-borne and bloodstream parasites, have implicated members of the gp85/trans-sialidase superfamily, MT gp82 and TCT Tc85-11, in cell invasion and interaction with host factors. Here we analyzed the gp82 structure/function characteristics and compared them with those previously reported for Tc85-11. One of the gp82 sequences identified as a cell binding site consisted of an α-helix, which connects the N-terminal β-propeller domain to the C-terminal β-sandwich domain where the second binding site is nested. In the gp82 structure model, both sites were exposed at the surface. Unlike gp82, the Tc85-11 cell adhesion sites are located in the N-terminal β-propeller region. The gp82 sequence corresponding to the epitope for a monoclonal antibody that inhibits MT entry into target cells was exposed on the surface, upstream and contiguous to the α-helix. Located downstream and close to the α-helix was the gp82 gastric mucin binding site, which plays a central role in oral *T. cruzi* infection. The sequences equivalent to Tc85-11 laminin-binding sites, which have been associated with the parasite ability to overcome extracellular matrices and basal laminae, was poorly conserved in gp82, compatible with its reduced capacity to bind laminin. Our study indicates that gp82 is structurally suited for MT to initiate infection by the oral route, whereas Tc85-11, with its affinity for laminin, would facilitate the parasite dissemination through diverse organs and tissues.

## Introduction

Host cell invasion and dissemination within the host are required for many pathogenic microorganisms to establish infection. Different pathogens may employ common tactics as well particular strategies for interaction with host components and for cell invasion. Enteropathogenic bacteria rely on their ability to bind to mucins, the main component of the mucus layer that protects the gastrointestinal mucosa, in order to reach the target cells. *Shigella dysenteriae*, for instance, adheres preferentially to colonic mucin as the first step to gain access to the colonic epithelial cells, within which it replicates after invasion [1], [2]. *Helicobacter pylori*, which does not invade cells but attaches to gastric epithelial cells and causes ulcers, binds to human gastric mucin at acidic pH [3]. *Trypanosoma cruzi*, the protozoan parasite that causes Chagas disease, binds selectively to gastric mucin as a prelude to traverse the mucus layer towards the underlying target cells [4]. Microbial infection may be facilitated by binding to extracellular matrix components. An invasive *Escherichia coli* isolate was found to bind basement-membrane laminin as opposed to non-invasive *E. coli* that exhibited only low-level laminin binding [5]. Infection by *T. cruzi* was dramatically reduced by stable knock down of host cell laminin gamma-1 by RNA interference [6].

Studies with MT generated in vitro and tissue culture-derived trypomastigotes (TCT), as counterparts of insect-borne and bloodstream parasites, have revealed the MT stage-specific surface molecule gp82 and Tc85-11 expressed in TCT, which are members of the gp85/trans-sialidase superfamily, as key players in the process of cell invasion [7], [8]. Gp82 mediates MT invasion of host cells by inducing signaling cascades that culminate in lysosome exocytosis [9], an event required for parasite internalization [10], [11]. In vivo, gp82 plays a central role in the establishment of *T. cruzi* infection in mice by the oral route [12], a mode of transmission that has been responsible for frequent outbreaks of acute Chagas disease in recent years [13]–[19]. A property of gp82 critical for oral *T. cruzi* infection is its ability to bind to gastric mucin present in the mucus layer that protects the stomach mucosa [4]. It has been proposed that, upon binding to gastric mucin, MT migrate through the mucus layer and reach the underlying epithelial cells that they invade in a gp82-mediated manner [20]–[22]. In vitro, MT were found to efficiently translocate through a gastric mucin layer [23]. Whether TCT exhibit such an ability has yet to be examined. On the other hand, TCT express Tc85-11 that binds laminin, a property that may enable the parasite to traverse extracellular matrices and reach the target cells [24].

Here we analyzed the structural characteristics of MT gp82 and their relation with specific functions of gp82 in host cell invasion and in gastric mucin binding. In addition, the structural/functional properties of MT gp82 were compared to those reported for TCT Tc85-11.

## Methods

### Homology Modeling of gp82 Protein

For the modeling of gp82 protein, we selected as a template the high resolution crystal structure of inhibitor-bound *Trypanosoma rangeli* sialidase (PDB 1N1T), which is closely related *T. cruzi* trans-sialidase, [25]. The gp82 sequence (Genbank L14824), which exhibited >39% identity when aligned with *T. rangeli* sialidase, was modeled using YASARA software (www.yasara.org) based on *T. rangeli* sialidase structure obtained from the Protein Data Base (www.rcsb.org). The best model was prepared for energy minimization and all the hydrogen atoms and other missing atoms from the model were created. Parameters for the force field were obtained from YAMBER3 [26], the pKa values for Asp, Glu, His and Lys residues were predicted. Based on the pH 7.0, the protonation states were assigned according to convention: Asp and Glu were protonated if the predicted pKa was higher than the pH, His was protonated if the predicted pKa was higher than the pH and it did not accept a hydrogen bond, otherwise it was deprotonated, Cys was protonated, Lys was deprotonated if the predicted pKa was lower than the pH, Tyr and Arg were not modified (www.yasara.org). A simulation box was defined at 15 Å around all atoms of each macromolecular complexes, then it was filled with water molecules and Na/Cl counter ions, that were placed at the locations of the lowest/highest electrostatic potential, until the cell neutralization, and the requested NaCl concentration reached 0.9%. A short molecular dynamics (MD) simulation was performed for the solvent adjust, and water molecules were subsequently deleted until the water density reached 0.997 g/ml. A short steepest descent energy minimization was carried out until the maximum atom speed dropped below 2,200 m/s. Then 500 steps of simulated annealing were performed with a temperature 0 K. Finally, a 45 nanosecond simulation at 298 K and a non-bonded cutoff of 7.86 A was carried out. A snapshot was saved every 7.5 picosecond. The graphical analysis was carried out using Visual Molecular Dynamics (VMD) software [27]. The average structure based on the last 20 nanoseconds was submitted to an energy minimization and used in all analyses. Validation procedure using the program PROCHECK [28] demonstrated that the final 3D structure agreed with the distance restrains and offered good geometry and side chain packing. The residues’ exposure for the solvent was analyzed using the program DSSP [29].

### Production and Purification of Recombinant Protein J18

The recombinant protein J18, containing the full-length *T. cruzi* gp82 (GenBank L14824) in frame with gluthatione S-transferase (GST), was produced in *E. coli* DH5-α and purified as previously described [30].

### Binding of the Recombinant Protein J18 to Gastric Mucin or Laminin

Microtiter plates (96 wells) were coated with mucin from porcine stomach (Type III, Sigma) or with laminin in PBS (10 µg/well). The antibodies used to ascertain by ELISA the effective coating were: antibodies against gastric mucin, generated by immunizing mice with porcine gastric mucin as described [4], and rabbit antibodies to mouse laminin, kindly provided by Dr. José Daniel Lopez, Universidade Federal de São Paulo. For J18 binding assay, the microtiter plates coated with gastric mucin or laminin were blocked with PBS containing 2 mg/ml bovine serum albumin (PBS/BSA) for 1 h. The plates were sequentially incubated at 37°C for 1 h with the recombinant protein J18, and peroxidase-conjugated anti-mouse IgG, all diluted in PBS/BSA, and the final reaction was revealed by *o*-phenilenediamine and the absorbance at 490 nm was read in ELx800™ absorbance microplate reader (BioTek).

### Parasites

*T. cruzi* (Y strain) was maintained cyclically in mice and in liver infusion tryptose (LIT) medium containing 5% fetal bovine serum. To promote differentiation to metacyclic forms, the parasites were grown for one passage in Grace’s medium (Invitrogen). For MT purification, parasites harvested from cultures at the stationary growth phase were passed through DEAE-cellulose column, as described [31]. TCT were obtained as follows: Vero cells, purchased from Instituto Adolfo Lutz, São Paulo, Brazil, were infected with MT. Five to six days later, the trypomastigotes released into the medium were collected.

### Parasite Migration Assay through Gastric Mucin Layer

Polycarbonate transwell filters (3 µm pores, 6.5 mm diameter, Costar) were coated with 50 µl of a preparation containing 10 mg/ml gastric mucin in water. Parasites (MT or TCT) suspended in 600 µl PBS were added to the bottom of 24-well plates (1.0×10^7^ parasites/well). Mucin-coated transwell filters were placed onto parasite-containing wells, and 100 µl PBS were added to the filter chamber. At 30 and 60 min incubation at 37°C, 10 µl were collected from the filter chamber for determination of parasite number. Assays were also performed with transwell filters coated with 50 µl of a preparation containing gastric mucin (10 mg/ml) mixed with the recombinant protein J18 (1 mg/ml) or GST (1 mg/ml), or coated with 50 µl of a gastric mucin preparation (10 mg/ml) mixed with the 20-mer synthetic peptide p7 or p7* at. 1 mg/ml.

### Treatment of Parasites with Pepsin

Treatment of parasites with pepsin was performed under two conditions, one using sodium citrate 0.1 mM, pH 3.5, and the other using the same buffer plus 0.9% NaCl, at pH 3.5. Parasites were incubated with pepsin at 2 mg/ml in either of the citrate buffer, at 37°C for 30 min, and then examined in the light microscope.

### Statistical Analysis

The significance level of experimental data was calculated using the Student’s *t* test, as implemented in the program GraphPad InStat.

### Ethics Statement

All procedures and experiments conformed with the regulation of the Universidade Federal de São Paulo Ethical Committee, in accord with Resolution N° 196 (10/10/1996) of National Council of Health, and the study was approved by the Committee.

**Figure 1:**
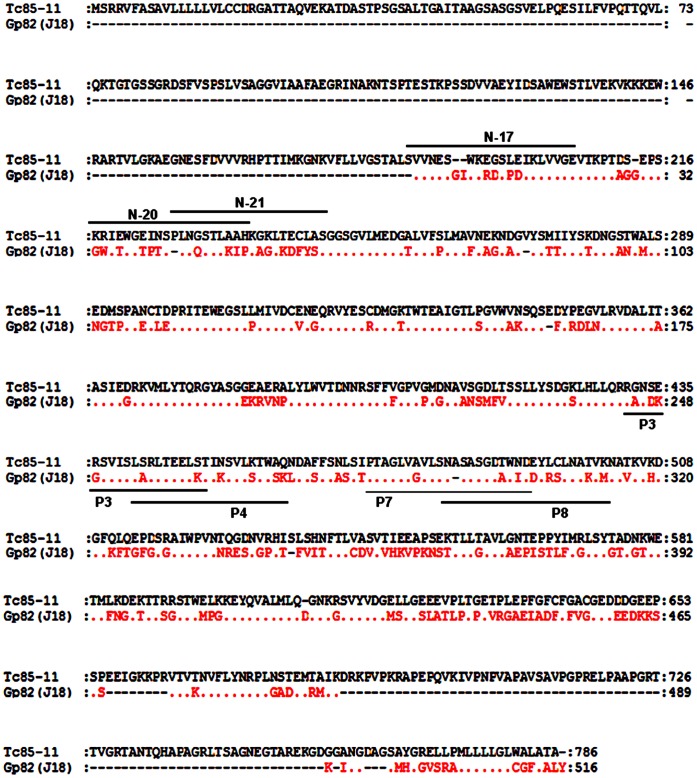
Sequences of *T. cruzi* surface proteins gp82 and Tc85-11. Shown are the aminoacid sequences deduced from cDNA clone J18, containing the full-length metacyclic stage gp82 (GenBank L14824), and from the cDNA insert containing Tc85-11 open reading frame (GenBank AF085686). In gp82, the sequences identified as P4 and P8 represent the host cell binding sites, P3 corresponds to the epitope for mAb 3F6, and P7 constitutes the main gastric mucin-binding site. In Tc85-11, the sequences corresponding to cell adhesion sites are identified as N-17, N-20 and N-21 and overlap with laminin-binding sites N17 and N-21 [34]. Points represent residues that are conserved in the two proteins, nonconserved amino acids are indicated, and dashes represent residues that are lacking.

**Figure 2:**
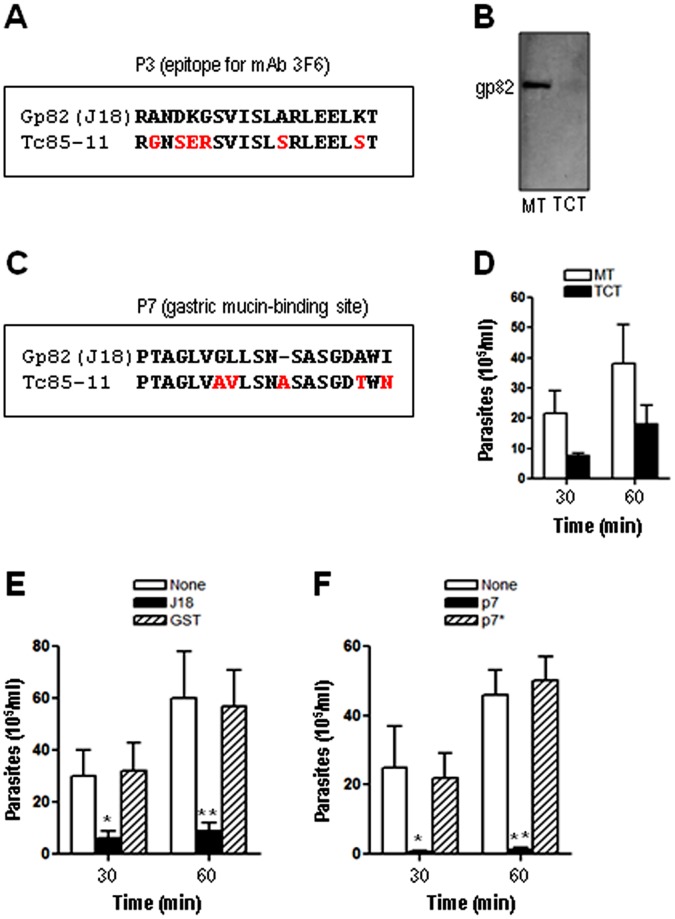
Comparison of gp82 sequences associated with recognition by mAb 3F6 or binding to gastric mucin with the equivalent sequences in Tc85-11. A) The gp82 sequence identified as the epitope for mAb 3F6 (P3) was aligned with the equivalent Tc85-11 sequence, with the differences highlighted in red. B) Soluble extracts of MT and TCT were analyzed by Western blot using mAb 3F6. C) The gp82 sequence corresponding to the gastric mucin-binding site (P7) was aligned with the equivalent Tc85-11 sequence, with the changed residues indicated in red. D) Transwell filters coated with gastric mucin were placed onto 24-well plates containing MT or TCT. After 30 or 60 min incubation, samples from the filter chamber were collected and the number of parasites counted. Values are the means ± SD of three independent experiments. E) Assays were performed as in (D) using transwell filters coated with gastric mucin alone, or mixed with the recombinant protein J18 or GST. The difference between the filter containing J18 and the control was significant (*P<0.05, **P<0.01). F) Assays were performed as in (D) using transwell filters coated with gastric mucin alone, or mixed with the synthetic peptide P7 or P7*. The difference between the filter containing P7 and the control was significant (*P<0.05, **P<0.0005).

**Figure 3:**
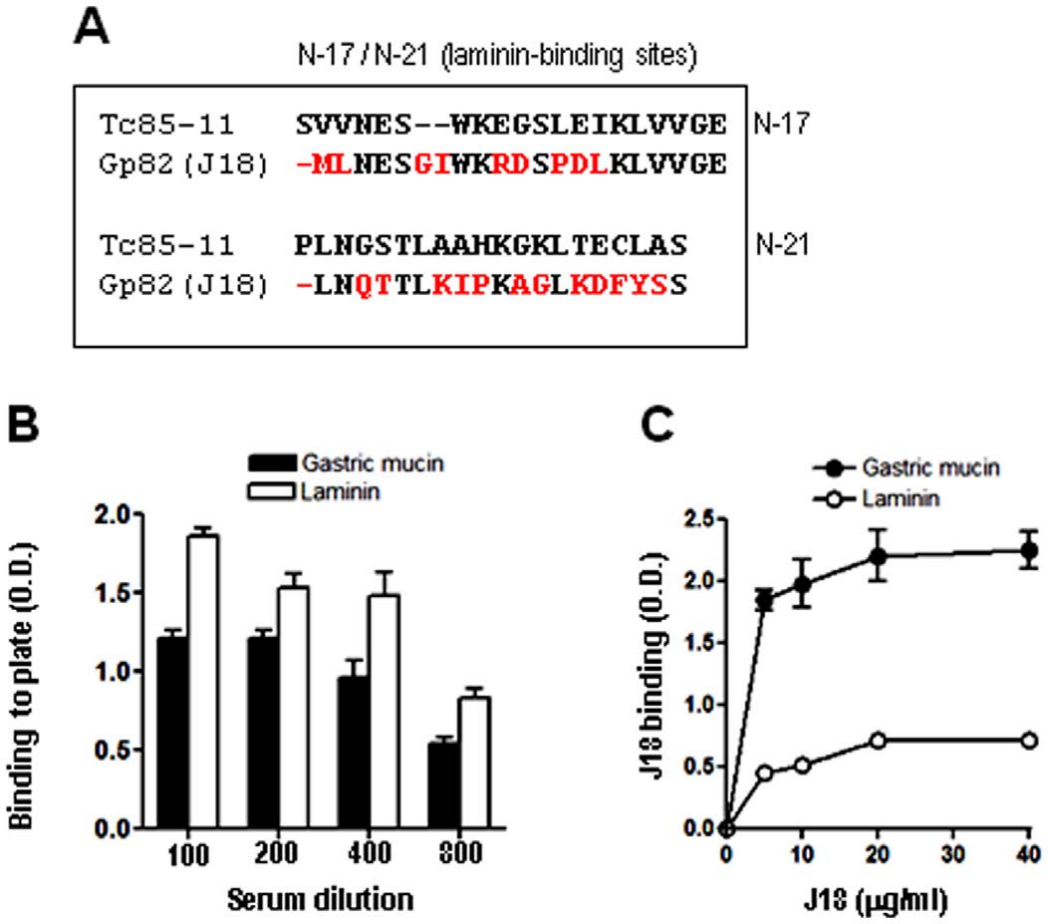
Comparative analysis of Tc85-11 sequences mapped as laminin-binding sites and the equivalent sequences in gp82. A) The Tc85-11 sequences N-17 and N-21, corresponding to laminin-binding sites, were aligned with the equivalent sequences in gp82, with the differences highlighted in red. B) Microtiter plates were coated with laminin or gastric mucin (10 µg/well), and ELISA assay was performed using anti-laminin or anti-gastric mucin antisera, at the indicated dilutions. C) Laminin- or gastric mucin-coated plates were incubated with J18, the recombinant protein containing the full length gp82 sequence, at the indicated concentrations. Binding of J18 was revealed by anti-J18 antibodies. Values are the means ± SD of triplicates of a representative experiment.

## Results

### Comparative Analysis of Sequences Relevant for T. Cruzi Infection in gp82 and Tc85-11 Proteins

In previous studies, the amino acid sequences deduced from a cDNA clone (J18) containing the full-length metacyclic stage gp82 (GenBank L14824) [32] and of a cDNA insert containing Tc85-11 open reading frame (GenBank AF085686) [24] have been reported. Here we compared the two sequences (Fig. 1), which share 54% identity and 64% similarity, focusing on sites considered to be important for *T. cruzi* infection. In gp82, the sequences P4 (amino acids 254–273) and P8 (amino acids 294–313) identified as the host cell adhesion sites [33] are localized in the C-terminal domain (Fig. 1). By contrast, the functionally equivalent sites in Tc85-11, represented by sequences N-17, N-20 and N-21 (Fig. 1), are present in the N-terminal domain [34]. The gp82 sequence P3 (amino acids 244–263), corresponding to the epitope for monoclonal antibody (mAb) 3F6 [33], which inhibits MT entry into host cells [35], partially overlaps the P4 sequence (Fig. 1). Compared to P3, the equivalent sequence in Tc85-11 exhibited considerable difference, with several non conservative amino acid substitutions (Fig. 2A) that may preclude its recognition by the referred antibody. No protein was revealed by mAb 3F6 in Western blot of TCT extract (Fig. 2B). P7 (amino acids 284–303), the sequence identified as the main gp82 gastric mucin-binding site that is associated with the MT capacity to migrate through the gastric mucin layer [4], had its counterpart in Tc85-11 and exhibited five amino acid substitutions, two of which were conservative (Fig. 2C). Assays were performed in which the ability of MT and TCT to traverse a gastric mucin layer was compared. The number of MT that traversed the mucin layer was about two-fold higher than TCT (Fig. 2D). Translocation of non infective epimastigotes, which do not express gp82 or Tc85-11, was about 25-fold lower as compared to MT. To further demonstrate that the ability of metacyclic forms to migrate through the gastric mucin layer was dependent on gp82, more specifically on P7 sequence, additional experiments were carried out. In one set, the transwell filters were coated with gastric mucin mixed with the recombinant protein J18, which is fused to GST, or with gastric mucin mixed with GST. Migration of metacyclic forms through the gastric mucin mixed with J18 was significantly reduced, whereas the presence of GST had no inihibitory effect (Fig. 2E). The other experiment consisted in coating the transwell filters with gastric mucin mixed with the synthetic peptide P7 or P7*. Peptide P7 corresponds to the gp82 gastric mucin binding site and was shown to inhibit the parasitism of gastric mucosal epithelium in oral *T. cruzi* infection [4]. Peptide P7* (LADLAGWLSPSDVGGAINST) has the same composition as P7 but with a scrambled sequence and is devoid of inhibitory effect on oral infection by metacyclic forms [4]. As shown in Fig. 2F, the presence of P7, but not of P7*, profoundly affected the parasite traversal through the gastric mucin.

**Figure 4:**
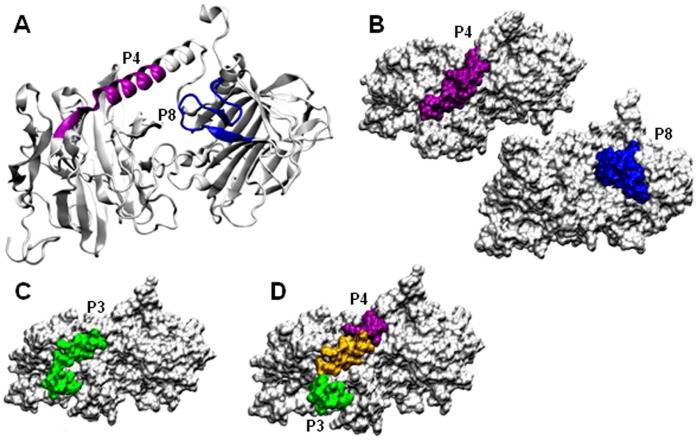
The structural model of gp82. A) Cartoon representation highlighting the cell binding sites P4 (magenta) and P8 (blue). B) Surface representation of sites P4 and P8. C) The epitope for mAb 3F6 (P3) is highlighted (green). D) The portion of P3 that overlaps with P4 is indicated (yellow).

**Table 1:** The solvent accessibility of the residues from peptides P4 and P8, measured in water exposed surface in Å^2^.

P4			P8		
Residue	AminoAcid	SolventAccessibility	Residue	AminoAcid	SolventAccessibility
254	L	67	294	N	66
255	A	32	295	S	57
256	R	129	296	A	15
257	L	9	297	S	47
258	T	67	298	G	56
259	E	111	299	D	95
260	E	54	300	A	18
261	L	7	301	W	4
262	K	122	302	I	39
263	T	64	303	D	0
264	I	0	304	D	47
265	K	82	305	Y	3
266	S	64	306	R	107
267	V	31	307	S	24
268	L	0	308	V	2
269	S	37	309	N	70
270	T	67	310	A	2
271	W	9	311	K	111
272	S	40	312	V	8
273	K	157	313	M	56

**Table 2:** The solvent accessibility of the residues from peptides P3 and P7, measured in water exposed surface in Å^2^.

P3			P7		
Residue	AminoAcid	SolventAccessibility	Residue	AminoAcid	SolventAccessibility
244	R	91	284	P	18
245	A	71	285	T	29
246	N	111	286	A	64
247	D	140	287	G	13
248	K	177	288	L	0
249	G	13	289	V	9
250	S	91	290	G	1
251	V	86	291	L	18
252	I	87	292	L	14
253	S	48	293	S	19
254	L	67	294	N	66
255	A	32	295	S	57
256	R	129	296	A	15
257	L	9	297	S	47
258	T	67	298	G	56
259	E	111	299	D	95
260	E	54	300	A	18
261	L	7	301	W	4
262	K	122	302	I	39
263	T	64	303	D	0

**Figure 5:**
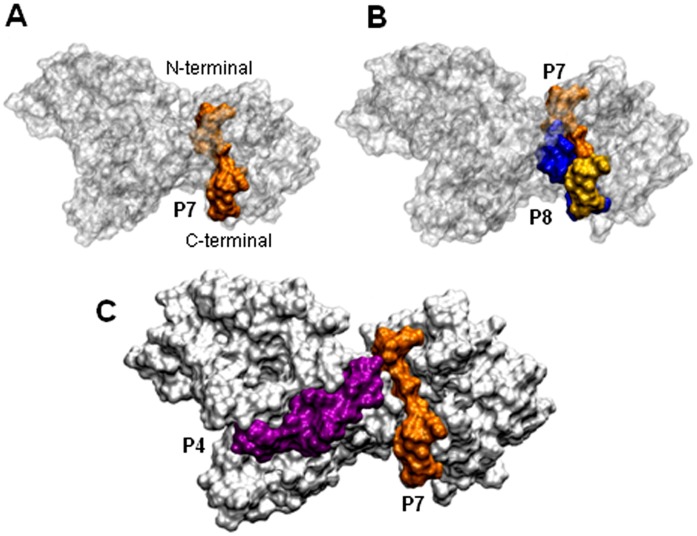
Surface representation of gp82 showing the gastric mucin-binding site. A) P7, the gastric mucin-binding site is highlighted in orange. B) Shown is the site P7 (orange) overlapped (yellow) with the cell binding site P8 (blue). C) The relative localization of P7 (orange) and the cell binding site P4 (magenta).

Tc85-11 is a laminin-binding protein [24]. Laminin-binding sites of Tc85-11have been mapped to sequences N-17 and N-21 (Fig. 1) that are nested within the target cell adhesion domain [34]. Equivalent sequences in gp82 exhibited marked differences, such as gaps either in J18 or Tc85-11, in addition to non-conservative amino acid substitutions (Fig. 3A), which may interfere with laminin-binding capacity. Assays to address this question were performed using microtiter plates coated with laminin, or gastric mucin as control. First, the effective coating of plates with these compounds was ascertained using antibodies to laminin or gastric mucin. The reactivity of serum containing anti-laminin and anti-gastric mucin antibodies with laminin and gastric mucin decreased with the increasing serum dilution (Fig. 3B), what assured us that we could use these coated plates to compare the J18 binding capacity. Next, the recombinant protein J18 containing the full-length gp82 sequence, at varying concentrations, was added to plates coated with laminin or gastric mucin, and the binding was revealed with anti-J18 antibodies. The ability of J18 to bind laminin was lower than its capacity to bind gastric mucin (Fig. 3C).

### Modeling of Metacyclic Form gp82 Protein

To obtain the structure model of MT gp82, we employed homology modeling, the same technique reported for obtaining the three-dimensional structure of Tc85-11. By using as templates the crystal structure of *T. cruzi* trans-sialidase and *T. rangeli* sialidase, which are closely related [25], [36], Tc85-11 was found to consist of a β-propeller domain at the N-terminal region connected by an α-helix joint to a β-sandwich domain at the C-terminal region [34]. Here, the high resolution crystal structure of inhibitor-bound *T. rangeli* sialidase (PDB 1N1T) was used as template. The model of gp82 was submitted to a molecular dynamics, and after 15 ns was stable (Fig. S1). The average model was analyzed with PROCHECK and all the stereochemical parameters were inside or better than those provided. In that model, the host cell binding site P4 was found to be part of the α-helix whereas P8 was located in the β-sandwich domain (Fig. 4A). Both P4 and P8 were exposed at the gp82 surface (Fig. 4B), compatible with the ability of these sequences to interact with host cells [33]. In P4, five amino acids with charged polar side chains had high solvent accessibility, whereas in P8 three amino acids were highly solvent-accessible (Table 1). Along the P3 sequence that corresponds to the epitope for mAb 3F6, nine residues displayed high solvent accessibility (Table 2). The almost full exposure of P3 (Fig. 4C) is in accord with the intense reaction of mAb 3F6 with live MT surface (Fig. S2). By binding to P3, which partially overlaps with the cell binding site P4 (Fig. 1 and 4D), mAb 3F6 possibly impairs parasite adhesion/invasion. Compared to the cell binding sites, the gastric mucin binding site represented by sequence P7 contained fewer solvent-accessible amino acids (Table 2) and was less exposed at gp82 surface (Fig. 5A). More specifically, its C-terminal portion that overlaps with the N-terminal sequence of P8 was exposed, whereas the more hydrophobic portion was buried (Fig. 5B). The surface representation of P7 in relation to the cell binding site P4, shown in Fig. 5C, suggests the possibility of simultaneous binding of gp82 to gastric mucin and the host cell, through P7 and P4, respectively. As concerns the VTV motif (VTVxNVxLYNR), highly conserved in gp85/trans-sialidase superfamily [37], it contained many amino acids barely accessible to solvent and, accordingly, was mostly buried (Fig. S3).

### Differential Susceptibility of MT and TCT to Pepsin Digestion

MT gp82 resists degradation by pepsin at acidic pH in vitro [20] and is preserved upon contact with the gastric juice in oral infection. In addition, MT surface is covered with protease-resistant mucin-like molecules [38], which protect the parasites from lysis in the gastric milieu. As blood trypomastigotes have been reported to rarely infect mice by the oral route [39], we checked whether these parasite forms are susceptible to peptic digestion, using TCT. When treated with pepsin at pH 3.5 for 30 min, TCT was mostly lysed (>90%) whereas MT preserved their morphology and motility.

## Discussion

The metacyclic stage surface molecule gp82, which is highly conserved among genetically divergent *T. cruzi* lineages [40], plays a central role in the process of host cell invasion and in the establishment of infection by the oral route, through its cell adhesion and gastric mucin-binding properties [4], [7]. In this study, we have shown that the referred properties conform to the structural features of gp82. Both P4 and P8 sequences, identified as the host cell binding sites of gp82, were exposed. This should enable their recognition by the corresponding receptors. Of the two sites, P4 has been shown in diverse experiments to be the main cell binding site [33], [41]. As regards P3, the epitope for the monoclonal antibody 3F6, its partial overlap with P4 and the confirmation that it is exposed on the surface reinforced the notion that this antibody exerts its inhibitory effect on MT internalization by sterical hindrance of P4. The gp82 sequence P7, identified as the gastric mucin-binding site, was nested in the cell-binding domain. Whether this positioning is favorable to gp82 interaction with gastric mucin and subsequently with the target epithelial cells, is not known. We visualize one possible scenario. In oral infection, metacyclic forms bind to gastric mucin upon reaching the stomach and migrate toward the underlying gastric epithelial cells. The recognition of the gp82 sequence P4 by its target cell receptor would facilitate the P7 release from the gastric mucin, enabling the parasites to initiate cell invasion. As the proximity of P4 to P7 is not so close, this would allow the P4-mediated binding of gp82 to target cells, while still bound to gastric mucin. In this scenario, P8 would play a minor role because of its partial overlap with P7.

Distinct from MT, blood trypomastigotes are inefficient in infecting mice by the oral route [39]. This could be due to the differential capacity of the two infective forms in migrating through the gastric mucin layer. When that possibility was tested using TCT, it was found that this parasite form traversed the mucin layer at two-fold lower numbers than MT, a result compatible with the observation that, as compared to the gp82 sequence P7, the equivalent sequence in Tc85-11 protein displayed a few non-conservative amino acid substitutions. Expression of gp82 or Tc85-11 on the parasite surface is presumably an important requirement for gastric mucin translocation. Epimastigotes that lack either of these molecules displayed very reduced ability to migrate through a gastric mucin layer, about 25 fold lower than MT. The difference between MT and TCT in traversing the gastric mucin layer did not seem to be sufficient to explain the low efficiency of blood trypomastigotes in infecting by the oral route. The possibility that blood trypomastigotes were more susceptible to pepsin digestion at acidic pH was tested using TCT. Lysis of TCT by pepsin at acidic pH was higher than 90% whereas MT preserved their morphology and motility, in the same manner as MT recovered from the mouse stomach 1 hour after oral infection [21]. If the blood trypomastigotes resisted peptic digestion, it is possible that they would be able to overcome the gastric mucin barrier.

Metacyclic forms interact with host cells and host components at the portal of entry, in the skin, the ocular mucosa or the stomach, whereas blood trypomastigotes have to overcome many barriers such as extracellular matrices and basal laminae to reach the target cells. In this context, the interaction of parasites with these components is critical for the dissemination within the host, and such a requirement is possibly fulfilled by the expression of Tc85-11, with its laminin-binding property [24], and/or by an 85 kDa protein that interacts with cells bearing fibronectin molecules [42]. It is of interest that the Tc85-11 sequences mapped as laminin-binding sites were nested in the cell adhesion domain [34]. Away from the laminin-binding sites of Tc85-11, in the C-terminal portion, lied the conserved VTV motif, whose function is not known. By using a synthetic peptide based in VTV motif, it was shown that it binds to cytokeratin 18 [43]. But this finding probably does not bear any association with the recognition of Tc85-11 by cytokeratin 18 and TCT entry into host cells. Transient silencing of cyotkeratin 18 gene in RNAi-treated HeLa cells did not affect binding and invasion of TCT [44]. Furthermore, a recombinant protein based on amastigote surface protein-2 containing the VTV motif failed to bind cytokeratin 18 [44]. In gp82, the VTV motif localized very close to the hydrophobic sequence that putatively is replaced by GPI, at the C-terminal end. This localization, and the fact that VTV motif is mostly unexposed, makes its interaction with host cell or with host factors unlikely.

In summary, metacyclic forms and blood trypomastigotes appear to use very closely related surface molecules to interact with distinct host components that they would find in natural infection, in order to reach the target cells and to ascertain their survival within the host.

## Supporting Information

Figure S1 **The α-carbon Root Mean Square Deviation from the gp82 model plotted as a function of simulation time.** A small oscillation was observed during the first 15 ns and after that the model became stable.
(TIF)

Figure S2 **Reaction of** ***T. cruzi*** **metacyclic trypomastigotes with mAb 3F6.** Live parasites were incubated with mAb 3F6 and processed for visualization at fluorescence microscope.
(TIF)

Figure S3 **The MT gp82 VTV motif.** A) VTV motifs of gp82 and Tc85-11 are aligned. B) Surface representation of gp82 VTV motif.
(TIF)
