# Supplementary figures and images for: Structural Basis of the Interaction of a Trypanosoma cruzi Surface Molecule Implicated in Oral Infection with Host Cells and Gastric Mucin
Source: PLoS One. 2012 Jul 31;7(7):e42153. doi: 10.1371/journal.pone.0042153 (PMC3409152; doi:10.1371/journal.pone.0042153)

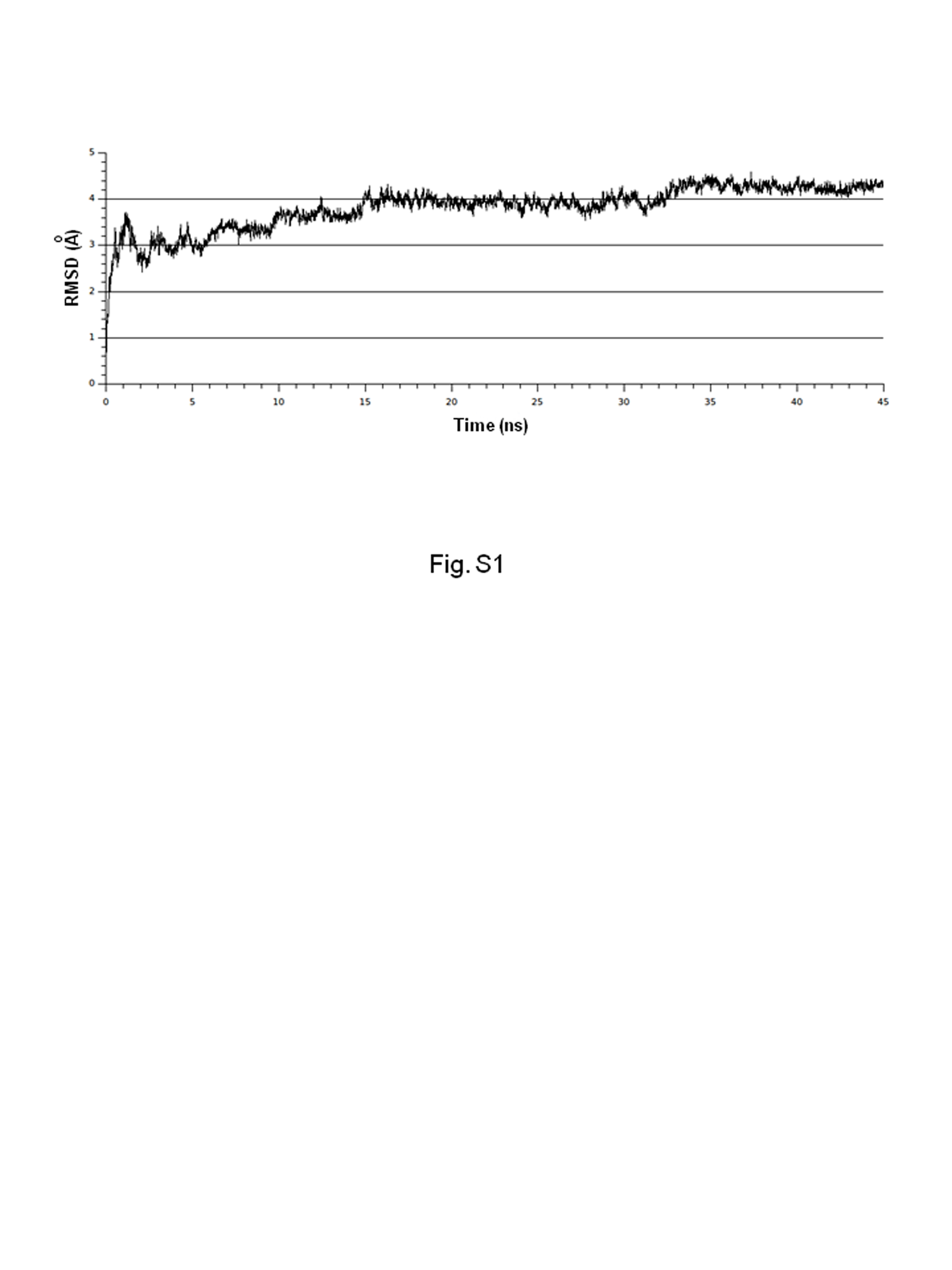

Supplement: Figure S1 — The α-carbon Root Mean Square Deviation from the gp82 model plotted as a function of simulation time. A small oscillation was observed during the first 15 ns and after that the model became stable. (TIF) [file pone.0042153.s001.tif]

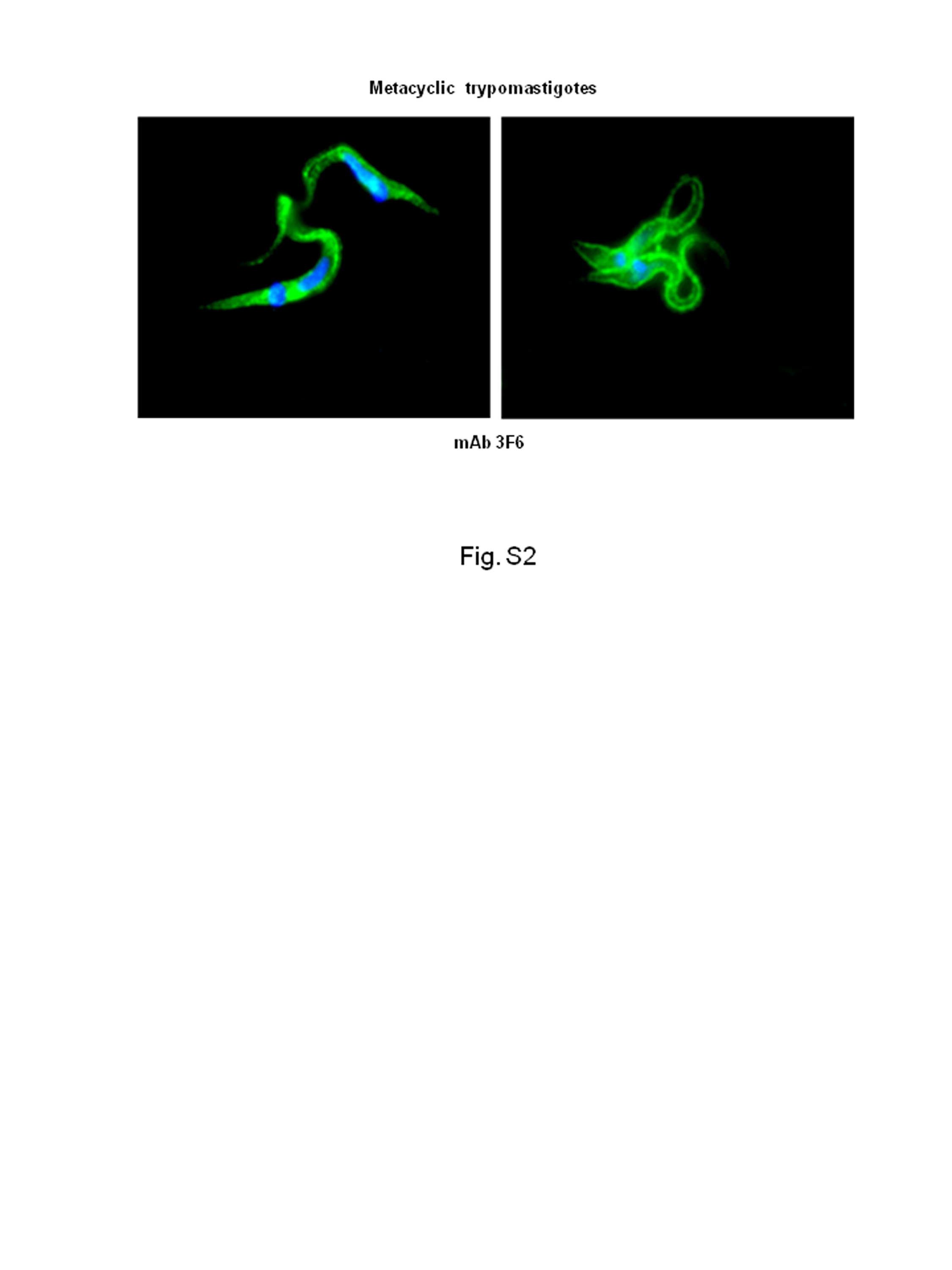

Supplement: Figure S2 — Reaction of T. cruzi metacyclic trypomastigotes with mAb 3F6. Live parasites were incubated with mAb 3F6 and processed for visualization at fluorescence microscope. (TIF) [file pone.0042153.s002.tif]

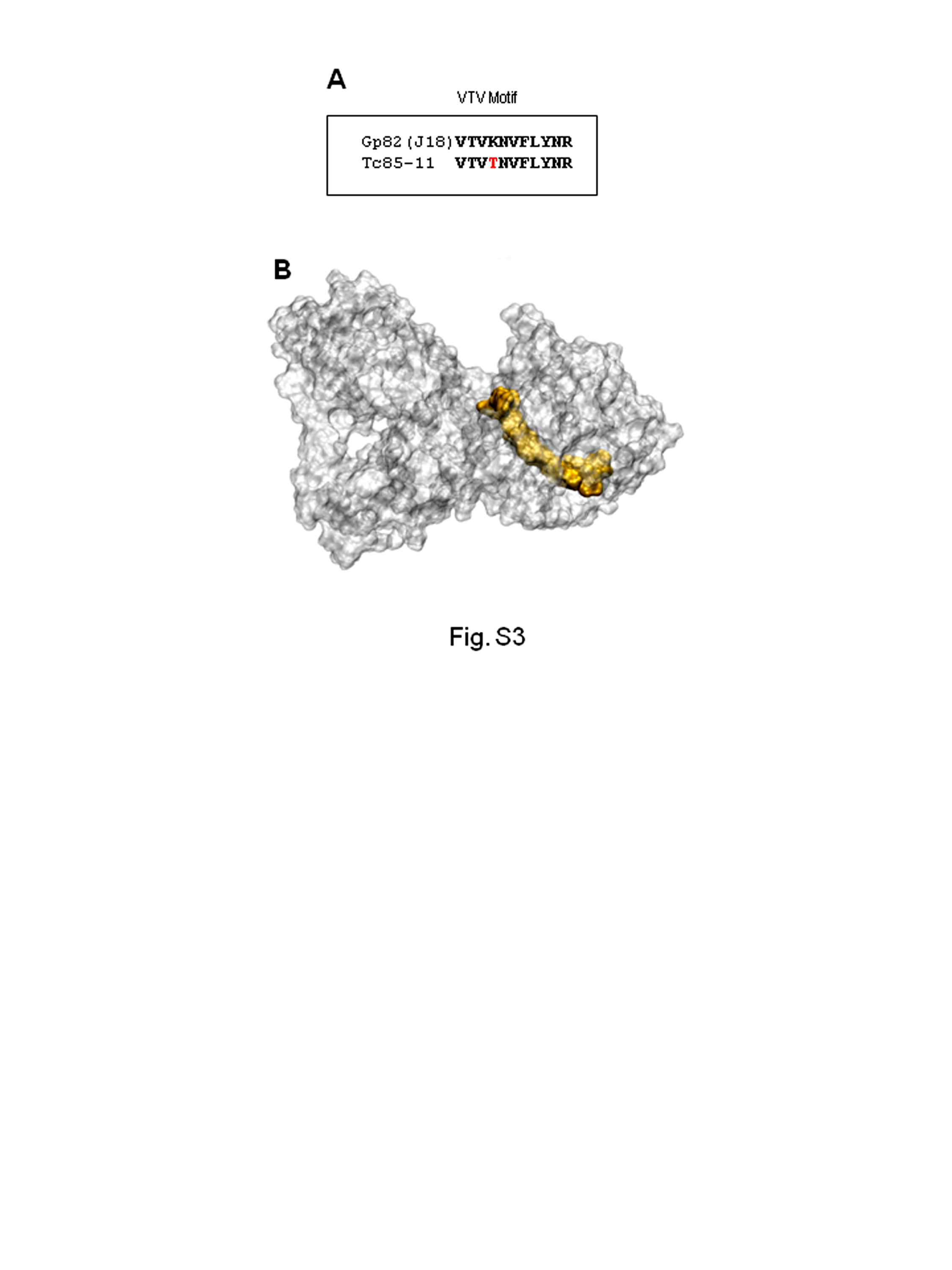

Supplement: Figure S3 — The MT gp82 VTV motif. A) VTV motifs of gp82 and Tc85-11 are aligned. B) Surface representation of gp82 VTV motif. (TIF) [file pone.0042153.s003.tif]
